# Altered frequency of peripheral B‐cell subsets and their correlation with disease activity in patients with systemic lupus erythematosus: A comprehensive analysis

**DOI:** 10.1111/jcmm.15836

**Published:** 2020-09-12

**Authors:** Yanxia Peng, Fengbiao Guo, Shuzhen Liao, Huanjin Liao, Haiyan Xiao, Lawei Yang, Hua‐feng Liu, Qingjun Pan

**Affiliations:** ^1^ Key Laboratory of Prevention and Management of Chronic Kidney Disease of Zhanjiang City Institute of Nephrology Affiliated Hospital of Guangdong Medical University Zhanjiang China; ^2^ Central Laboratory and Department of Neurology Shunde Hospital Southern Medical University (The First People’s Hospital of Shunde Foshan) Foshan China; ^3^ Department of Histology and Embryology Shantou University Medical College Shantou China; ^4^ Department of Laboratory Medicine Shanghai General Hospital Shanghai Jiao Tong University School of Medicine Shanghai China; ^5^ Department of Cellular Biology and Anatomy Augusta University Augusta GA USA

**Keywords:** B cell, flow cytometry, memory B cell, plasmablast, systemic lupus erythematosus

## Abstract

Alternations of peripheral B‐cell subsets are closely related to disease activity in systemic lupus erythematosus (SLE) and may also predict the relapse of SLE. In this study, we aimed to comprehensively analyse the frequency of peripheral B‐cell subsets, and their correlation with disease activity in patients with SLE. The results showed that for B‐cell subsets in the antigen‐independent differentiation stage, the frequency of the peripheral hematopoietic stem cell (HSC) subset in all patients with SLE was significantly higher than that of control patients. Surprisingly, several significant correlations were noted in newly diagnosed patients with SLE including a positive correlation in the frequency of the common lymphoid progenitor cell (CLP) with cholesterol serum levels. For B‐cell subsets in the antigen‐dependent differentiation stage, the frequency of naïve B‐cell (N‐B) subsets in all patients with SLE was significantly higher than that in the control patients. Moreover, the frequency of plasmablasts positively correlated with the SLEDAI score in the newly diagnosed patients. For memory B‐cell (M‐B) subtypes in the antigen‐dependent differentiation stage, the frequency of the class‐switched memory B‐cell (CSM‐B) subsets was positively correlated with the serum levels of complement C3. Notably, the frequency of the CSM‐B subset also negatively correlated with the SLEDAI score, whereas the non–class‐switched memory B‐cell (NSM‐B) subset was positively correlated with the serum levels of haemoglobin. Collectively, these findings may contribute to a better understanding of the role played by different B‐cell subsets in the pathogenesis of SLE.

## 1 INTRODUCTION

Systemic lupus erythematosus (SLE) is a complex systemic autoimmune disease that affects nearly all organs, including the skin, joints, kidneys and central nervous system.^1^, ^2^ The incidence of SLE is approximately 1‐10/100 000/year, its prevalence approximately 20‐70/100,000, and the disease often occurs in women of childbearing age.^3^ SLE is characterized by the production of large amounts of autoantibodies, forming autoantigen immune complexes that are deposited in local tissues, inducing inflammatory reactions and tissue damage.^4^, ^5^

B cells are known to play a key role in the development of SLE.^6^ Moreover, the role of hyperactivated B cells in the production of autoantibodies and inflammatory cytokines is well established.^4^, ^5^ B‐cell hyperproliferation and autoimmune B‐cell clearance disorders lead to the production of a variety of autoantibodies,^7^ causing systemic inflammation and organ damage.^8^ In addition, activated B cells can also accelerate the SLE pathogenesis by secreting inflammatory cytokines such as IL‐6, IL‐10, IL‐12 and TNF‐α.^9^, ^10^ Studies have shown that the targeting of B cells may result in a good therapeutic effect in SLE,^11^, ^12^ which further proves the importance of B cells in SLE pathogenesis.

Depending on their developmental phase, B cells are divided into two stages, namely the antigen‐independent differentiation and antigen‐dependent differentiation stages. B‐cell development is a highly regulated process that varies depending on the microenvironment.^13^, ^14^ Early B‐cell differentiation occurs in the bone marrow, while mature B cells, memory B cells (M‐Bs) and plasmablasts develop in the peripheral lymphoid organs such as the lymph nodes and spleen. Based on the expression of different membrane markers, including CD10, CD19, CD27, CD38, CD34, IgD and IgM, B cells can be divided into several different subsets. Normal B‐cell development contributes to the maintenance of humoral immunity in the body. Abnormalities in the development or differentiation of B cells, as well as in their activation, may lead to various immune system disorders and autoimmune diseases, such as SLE, rheumatoid arthritis and tumours.^7^, ^15^, ^16^, ^17^

Notably, alternations of peripheral B‐cell subsets are closely related to disease activity in SLE and may also predict patient relapse. According to CD5 expression, B cells are distinguished into B1 (CD5^+^) and B2 (CD5^‐^) subpopulations.^18^ B1 cells exert anti‐infectious effects by participating in non‐specific immunity and also produce a variety of reactive autoantibodies.^19^, ^20^ B2 cells play a major role in antigen presentation, mediating specific humoral immune responses and immune regulation. In an attempt to analyse SLE pathogenesis, a previous study focused on the origins of the B1 or B2 antibody subpopulation.^21^ However, neither B1 nor B2 cells account entirely for the frequency levels of the B‐cell subsets; furthermore, most studies on lymphocyte subsets in the peripheral blood of patients with SLE have focused on the frequency of T‐cell subsets.^22^, ^23^, ^24^, ^25^

In this study, we comprehensively evaluated the altered frequency of peripheral B‐cell subsets, and their correlation with disease activity in patients with SLE. These findings may contribute to a better understanding of the role played by different B‐cell subsets in the pathogenesis of SLE.

## 2 MATERIALS AND METHODS

### 2.1 Patients and clinical parameters

A total of 81 patients with SLE were enrolled in this study at the Department of Nephrology of Affiliated Hospital of Guangdong Medical University between July 2017 and February 2019, in accordance with the modified SLE classification criteria formulated in 1997 by the American College of Rheumatology (ACR).^26^ Moreover, 39 healthy volunteers were enrolled as the control patients. Patients newly diagnosed with SLE (n = 48) had not received glucocorticoids or other immunosuppressive drugs, whereas the other group of patients (n = 33) with a disease duration of 4.3 ± 2.7 years (mean ± standard deviation [SD]) were under treatment. Patients with allergies, hepatitis B, cancer, and other severe systemic diseases or autoimmune disorders were excluded from this study. The two patient groups did not significantly differ in terms of sex or age. SLE was assessed according to the Systemic Lupus Erythematosus Disease Activity Index (SLEDAI) 2000.^27^ In each patient, the haemoglobin, serum albumin, serum creatinine, blood uric acid, cholesterol, serum complement C3 and C4, anti‐dsDNA IgG, and SLEDAI score were determined. Table 1 summarizes patients’ age, gender, positive rate anti‐dsDNA IgG and anti‐nuclear IgG, SLEDAI score, and therapy.

**TABLE 1:** Demographic characteristics of controls and patients with SLE

Demographic variables	Controls (n = 39)	SLE patients		
		Newly diagnosed (n = 48)	Treated (n = 33)	Total (n = 81)
Age				
Range, years	19‐47	18‐53	18‐56	18‐56
Mean, SD	27.1 ± 7.5	34.6 ± 15.7	36.2 ± 14.7	35.8 ± 14.2
Gender				
F/M, no. (%)	30 (76.9%)/9 (23.1%)	36 (75%)/12 (25%)	30 (90.9%)/3 (9.1%)	66 (81.5%)/15 (18.5%)
Anti‐dsDNA				
IgG positive: no. (%)	No.	33 (68.8%)	12 (36.4%)	45 (55.6%)
Anti‐nuclear				
IgG positive: no. (%)	No.	45 (93.8%)	32 (96.97%)	77 (95.06%)
SLEDAI score				
Mean, SD	No.	15.56 ± 4.59	7.09 ± 8.31	12.11 ± 7.53
Median (Min, Max)	No.	16 (11, 26)	4 (0, 28)	13 (0, 28)
Therapy				
Low‐dose corticosteroid	No.	No.	9 (27.3%)	9 (11.1%)
High‐dose corticosteroid	No.	No.	3 (9.1%)	3 (3.7%)
Immunosuppressant + corticosteroid	No.	No.	21 (63.6%)	21 (25.9%)

The study was approved by the ethics committee of our hospital. Written informed consent was obtained from all patients and control patients.

### 2.2 Flow cytometry analysis

Blood was collected from patients with SLE and control patients. The blood was then lysed with red blood cell lysis buffer (BD Biosciences, San Jose, USA) and centrifuged at 300 *g* for 5 min at 4℃, and the precipitation was washed twice. The supernatant was discarded, and the cells were resuspended in staining buffer (BD Biosciences, San Jose, USA). For B‐cell surface markers staining, cells were incubated for 30 min at room temperature with fluorochrome‐conjugated antibodies to cell‐surface molecules (PE‐mouse‐anti‐human CD10, APC‐mouse‐anti‐human IgM, FITC‐mouse‐anti‐human CD19, PE‐Cy^TM^7‐mouse‐anti‐human CD27, PE‐Cy^TM^7‐mouse‐anti‐human CD34, APC‐mouse‐anti‐human CD38 and PerCP‐Cy^TM^5.5‐mouse‐anti‐human IgD were purchased from BD Pharmingen^TM^ (USA). The cells were washed twice with phosphate buffered saline, and the expression of surface molecules was assayed using a Becton Dickinson FACS Canto II (BD Biosciences, San Jose, USA) flow cytometer. The data were analysed using FlowJo 10 software (Tree Star, Inc, Ashland, OR, USA).

To identify cell populations in the antigen‐independent differentiation stage, cell surface staining of CD34, CD10, CD19 and IgM was performed. B cells were divided into hematopoietic stem cell (HSC) (CD34^+^CD10^‐^CD19^‐^IgM^‐^), common lymphoid progenitor cell (CLP) (CD34^+^CD10^+^CD19^‐^IgM^‐^), progenitor B‐cell (Pro‐B) (CD34^+^CD10^+^CD19^+^IgM^‐^), precursor B‐cell (Pre‐B) (CD34^‐^CD10^+^CD19^+^IgM^‐^) and immature B‐cell (Im‐B) (CD34^‐^CD10^+^CD19^+^IgM^+^) subsets.

To identify cell populations in the antigen‐dependent differentiation stage, cell surface staining of CD19, CD10, CD27 and CD38 was performed. Im‐B (CD19^+^CD10^+^CD27^‐^CD38^+^), naïve B cell (N‐B) (CD19^+^CD10^‐^CD27^‐^CD38^+^), M‐B (CD19^+^CD10^‐^CD27^+^CD38^‐^) and plasmablast (CD19^+^CD27^hi^CD38^hi^) subsets were evaluated. Additionally, to identify M‐B populations in the antigen‐dependent differentiation stage, the expression of cell surface markers CD19, CD27 and IgD was evaluated to detect non‐class‐switched M‐B (NSM‐B) (CD19^+^CD27^+^IgD^+^), class‐switched M‐B (CSM‐B) (CD19^+^CD27^+^IgD^‐^) and double‐negative M‐B (DN‐B) (CD19^+^CD27^‐^IgD^‐^).

### 2.3 Statistical analysis

All data were analysed using SPSS statistics software (version 20.0; IBM Corp., Armonk, NY, USA) and expressed as mean ± SD or median [range] according to their distribution. Two‐group comparisons were performed using unpaired two‐tailed Student's *t* test. Multiple‐group comparisons were performed using one‐way analysis of variance, followed by least significant difference or Dunnett's T3 post hoc tests. The proportions of B‐cell subsets and the clinical parameters in patients with SLE were evaluated by Spearman correlation as a similarity metric and were represented as a heat map.

## 3 RESULTS

### 3.1 Frequency of B cells in the antigen‐independent differentiation stage in peripheral blood

**FIGURE 1:**
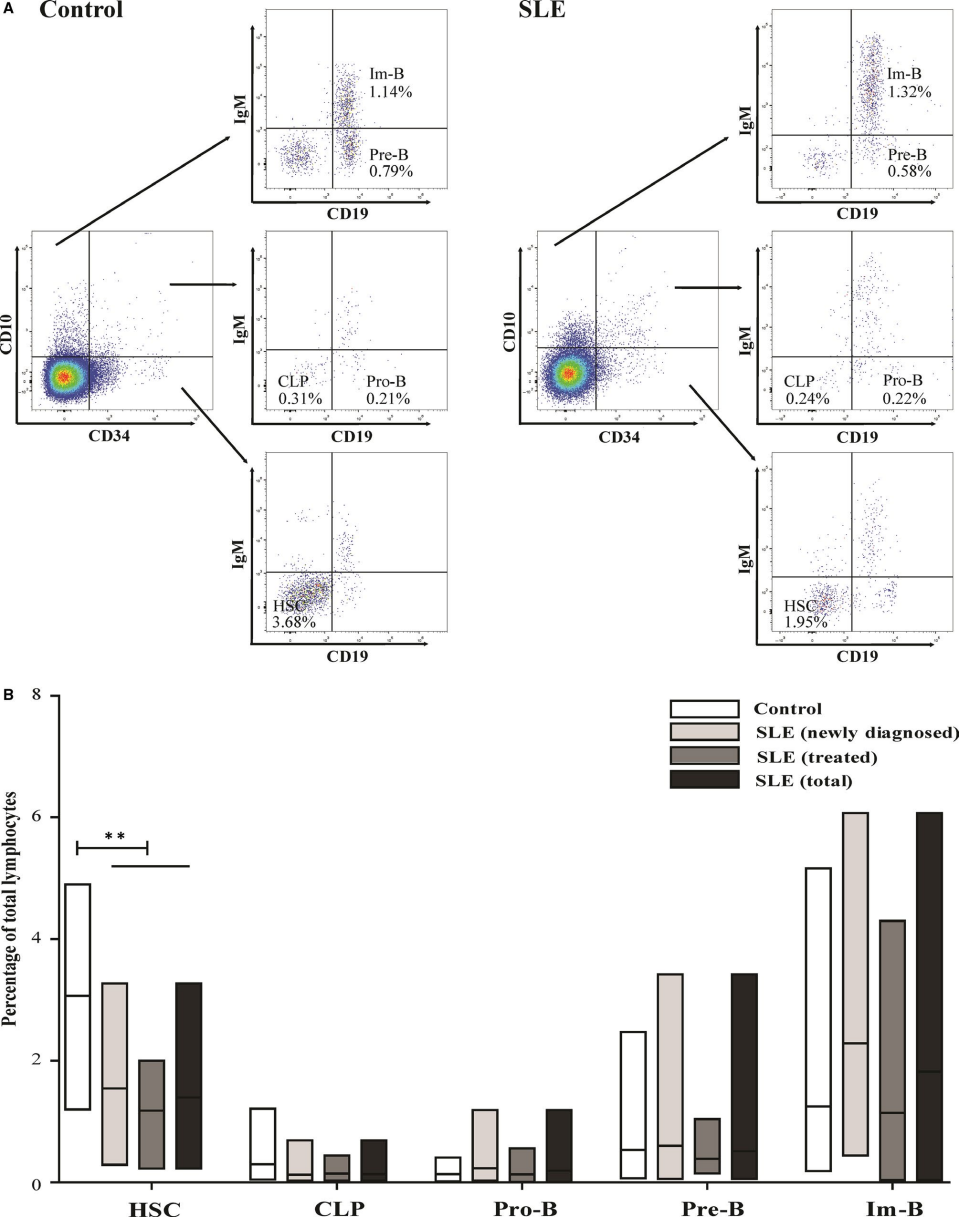
The frequency of B‐cell substages at antigen‐independent differentiation stage in the peripheral blood of normal controls (n = 39) and patients with SLE (n = 81). A, Representative plots of cell subsets in controls and patients with SLE. B, Statistical evaluation of the percentage of HSC, CLP, Pro‐B, Pre‐B and Im‐B populations in total lymphocytes of controls and patients with SLE. Data are expressed as mean [range]. ^*^ *P* < 0.05, ^**^ *P* < 0.01, ^***^ *P* < 0.001. (HSC: hematopoietic stem cell; CLP: common lymphoid progenitor cell; Pro‐B: progenitor B cell; Pre‐B: precursor B cell; and Im‐B: immature B cell)

Early B‐cell differentiation in the bone marrow can be divided into five substages, HSC, CLP, Pro‐B, Pre‐B and Im‐B. Some of these cell populations may be released into the peripheral blood and migrate to peripheral lymphoid organs. However, the frequency of these B cells in the peripheral blood of patients with SLE is not fully elucidated. We first measured the frequency of B cells in the antigen‐independent differentiation stage in the peripheral blood of control patients and patients with SLE (Figure 1A). The results showed that the frequency (percentage of total lymphocytes) of peripheral HSC subsets significantly decreased in all three patient groups: newly diagnosed, treated and total patients, compared to that in the controls (all *P* < 0.01) (Figure 1B).

### 3.2 Frequency of B cells in the antigen‐dependent differentiation stage in peripheral blood

**FIGURE 2:**
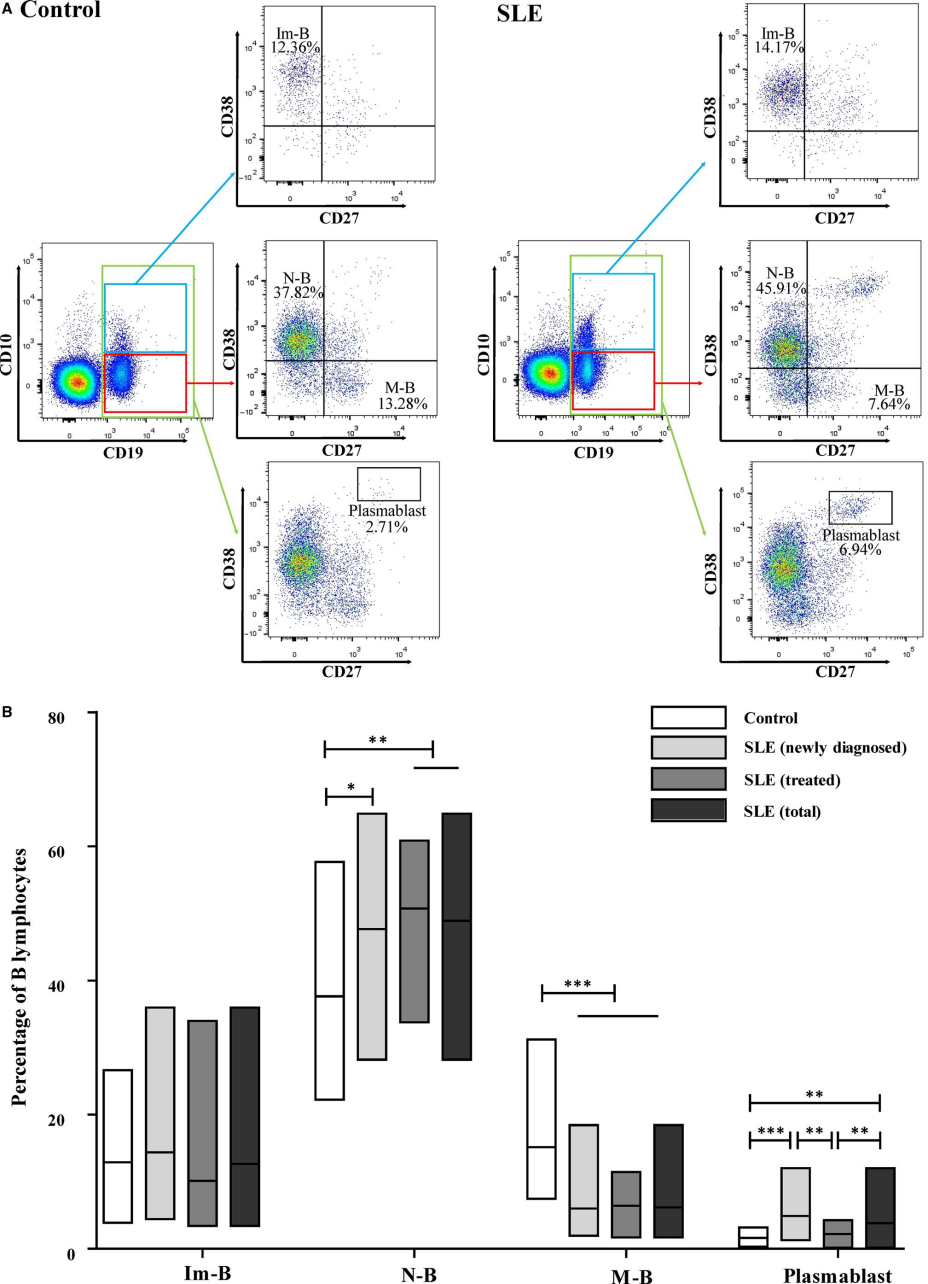
The frequency of B cell at antigen‐dependent differentiation stage in the peripheral blood of controls (n = 39) and patients with SLE (n = 81). A, Representative plots of cell subgroups in controls and patients with SLE. B, Statistical evaluation of the percentage of Im‐B, N‐B, M‐B and plasmablast populations in B lymphocytes of normal controls and patients with SLE. Data are expressed as mean [range]. ^*^ *P* < 0.05, ^**^ *P* < 0.01, ^***^ *P* < 0.001. (Im‐B: immature B cell; N‐B: naïve B cell; and M‐B: memory B cell)

Im‐Bs are released from the bone marrow into the peripheral blood and migrate to peripheral lymphoid organs, such as the spleen and lymph nodes. Here, they mature and finally differentiate into different subsets upon exposure to different foreign antigens. This process, also known as antigen‐dependent differentiation, involves Im‐Bs, N‐Bs, M‐Bs and plasmablasts. Subsequently, we measured the frequency of B cells in the antigen‐dependent differentiation stage in the peripheral blood of control patients and patients with SLE (Figure 2A). Here, we observed that the frequency of the N‐B subset significantly increased in patients with SLE compared to that in the control patients (for all patient groups, *P < *0.05) (Figure 2B), whereas the frequency of the M‐B subset significantly decreased in all patient groups (all *P* < 0.001) (Figure 2B). In addition, the frequency of the plasmablast subset significantly increased in both the newly diagnosed patients and total patient group (all *P* < 0.05), but not in the treated patients (Figure 2B) compared to that in the controls. Moreover, the frequency of the plasmablast subset was significantly higher in the newly diagnosed group than in the treatment group (*P* < 0.01) (Figure 2B).

### 3.3 Frequency of M‐Bs in the antigen‐dependent differentiation stage in peripheral blood

**FIGURE 3:**
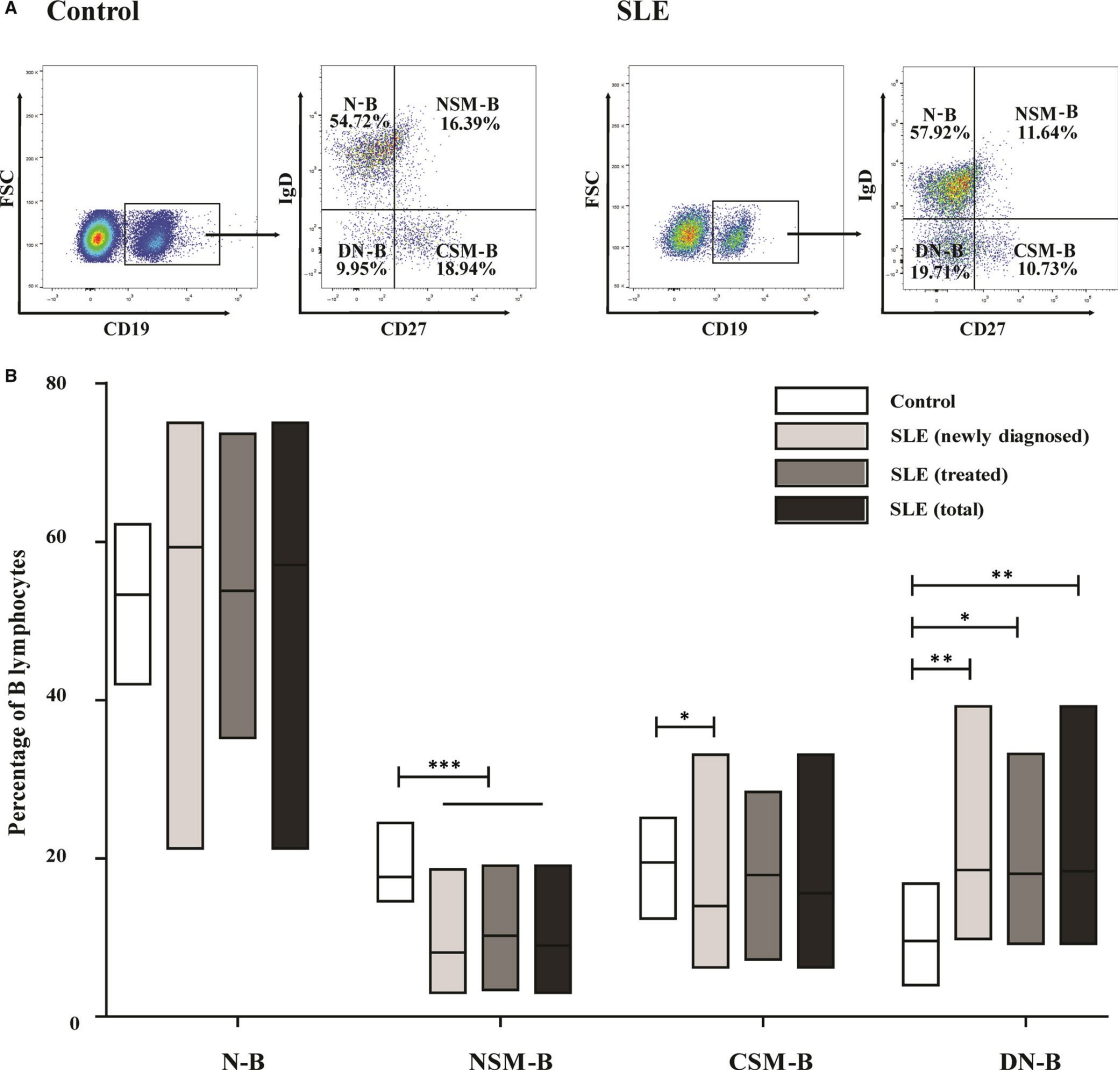
The frequency of memory B cell at antigen‐dependent differentiation stage in the peripheral blood of controls (n = 39) and patients with SLE (n = 81). A, Representative plots of cell subgroups in controls and patients with SLE. B, Statistical evaluation of the percentage of NSM‐B, CSM‐B, and DN‐B populations in B lymphocytes of controls and patients with SLE. Data are expressed as mean [range]. ^*^ *P* < 0.05, ^**^ *P* < 0.01, ^***^ *P* < 0.001. (M‐B: memory B cell; NSM‐B: non‐class‐switched memory B cell; CSM‐B: class‐switched memory B cell; and DN‐B: double‐negative memory B cell)

Upon exposure to foreign antigens, N‐Bs are known to differentiate into M‐Bs. This process can be divided into the following substages: N‐Bs, NSM‐Bs, CSM‐Bs and DN‐Bs. Herein, we evaluated the frequency of M‐Bs in the antigen‐dependent differentiation stage in the peripheral blood of control patients and patients with SLE (Figure 3A). The results showed that the frequency of the NSM‐B subset significantly decreased in all three patient groups (all *P* < 0.001) compared to that in the control patients (Figure 3B). Additionally, the CSM‐B frequency significantly increased in the newly diagnosed patients, but not in the two other patient groups, compared to that in the control patients (*P* < 0.05) (Figure 3B).

### 3.4 Correlation between B‐cell subsets and clinical parameters in newly diagnosed patients with SLE

**FIGURE 4:**
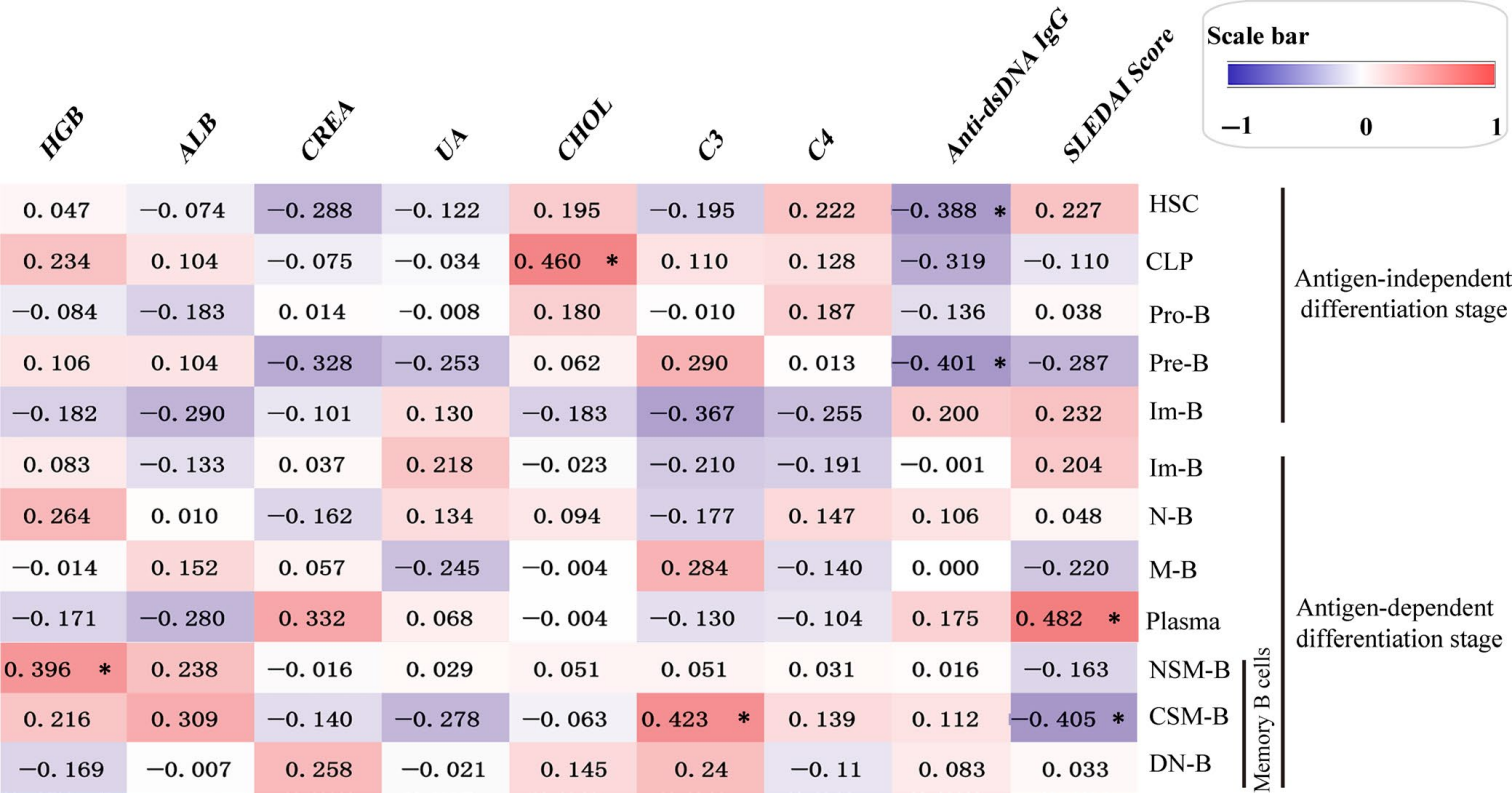
Spearman correlation analyses of B‐cell subsets at two differentiation stages and clinical parameters in newly diagnosed patients with SLE (n = 48). Red and blue colours represent positive and negative correlations, respectively. The colour intensity reflects the strength of the correlation (−1 to 1), as indicated by the scale bar. ^*^ *P* < 0.05, ^**^ *P* < 0.01, ^***^ *P* < 0.001. (HGB, haemoglobin; ALB, albumin; CREA, creatinine; UA, uric acid; and CHOL: cholesterol)

The correlation between B‐cell subsets and the clinical parameters in newly diagnosed patients with SLE was analysed using the Spearman method. Surprisingly, the results showed that in the antigen‐independent differentiation stage, the frequency of both the HSC and Pre‐B subsets was negatively correlated with the serum levels of anti‐dsDNA IgG (*r* = −0.388, *P* = 0.046; *r* = −0.401, *P* = 0.038, respectively). The frequency of CLP was positively correlated with the serum levels of cholesterol (*r* = 0.461, *P* = 0.016) in patients with SLE (Figure 4).

In the antigen‐dependent differentiation stage, the frequency of the plasmablasts was positively correlated with the SLEDAI score (*r* = 0.482, *P* = 0.011) in patients with SLE (Figure 4). Furthermore, the frequency of the CSM‐B subsets was positively correlated with the serum levels of complement C3 (*r* = 0.423, *P* = 0.045). The frequency of the CSM‐B subset was negatively correlated with the SLEDAI score (*r* = −0.405, *P* = 0.036) and that of the NSM‐B subset was positively correlated with the serum levels of haemoglobin (*r* = 0.396, *P* = 0.041) (Figure 4).

## 4 DISCUSSION

SLE is a multifactorial, autoimmune disease involving multiple organs and is characterized by immune system disorders.^28^ While B cells are known to play an important role in the pathogenesis of SLE,^29^ we present here, to our knowledge for the first time, a comprehensive analysis of the altered frequency of peripheral B‐cell subsets. These findings, together with a correlation analysis of disease activity in patients with SLE, may contribute to an increased understanding of the different B‐cell subsets in SLE pathogenesis.

For B‐cell subsets in the antigen‐independent differentiation stage, the frequency of the CD34^+^CD10^‐^CD19^‐^IgM^‐^HSC subset significantly decreased in patients newly diagnosed with SLE, patients treated for SLE and total number of patients with SLE. Our results were consistent with those of previous studies showing a decreased number of circulating CD34^+^ B cells in patients with SLE.^30^, ^31^ Furthermore, patients with SLE have a high level of apoptotic circulating CD34^+^ HSCs,^32^, ^33^ which may be due to a high rate of apoptosis or the inability to adequately clear the apoptotic HSCs in these patients.^34^ Moreover, the proportion of apoptotic HSCs is high in the bone marrow of patients with SLE.^35^ Contrarily, Michelle *et al* ^36^ reported an increase in the number of CD34^+^ HSCs in patients with SLE. These discrepancies may reflect differences in the patient selection criteria.

After differentiation in the bone marrow, mature B cells undergo a negative selection allowing for the clearance of autoreactive B cells and move from the bone marrow into the peripheral blood circulation.^37^ However, some studies have shown that some Im‐Bs in the bone marrow may differentiate into transitional B cells and, subsequently, leave the bone marrow to reach the peripheral circulation.^38^ Im‐B or N‐B subsets may differentiate into M‐Bs or plasmablasts in the lymph nodes, lymphoid follicles, spleen and peripheral blood, thereby producing specific antibodies upon antigen exposure.

Within the B‐cell subsets in the antigen‐dependent differentiation stage, the proportions of the Im‐B, N‐B, M‐B and plasmablast subsets in patients with SLE were determined in this study. Previous studies have shown that the percentage of N‐Bs is lower in patients with SLE than in control patients.^39^, ^40^ Additionally, Clarke *et al* ^41^ reported that autophagy is mainly activated in the N‐Bs of patients with SLE and that autophagy inhibition prevents human B cells from differentiating into plasmablasts. This demonstrated a requirement for autophagy in B‐cell survival and differentiation, particularly during early development, and in the formation of plasmablasts. Based on the latter study, we have been suggested that, in patients with SLE, an increase in the number of N‐Bs could promote B‐cell differentiation into plasmablasts. Moreover, our results showed that the frequency of the M‐B subset decreased in the peripheral blood of patients with SLE. Studies have shown that the number of CD27^+^ M‐Bs is lower in patients with SLE than in control patients.^39^, ^40^ Arce *et al* ^22^ showed that the frequency of B cells (CD19^+^CD20^+^CD38^‐^) is significantly lower in patients with SLE than in normal patients. M‐B subsets (CD19^+^CD27^+^CD38^‐^) are considered a risk factor for autoimmunity because of their low activation threshold.^42^ The reduced frequency of the M‐B subset may be related to the production of a large amount of autoantibodies in SLE, because of the transformation of M‐Bs into plasmablasts. We demonstrated a high frequency of plasmablasts in the total and newly diagnosed patients with SLE, but not in the patients treated for SLE. The number of plasmablasts in the patients treated for SLE was significantly lower than that in the patients newly diagnosed with SLE. The frequency of the plasmablast and plasma cell subsets is dramatically increased in the peripheral blood of patients with SLE.^39^, ^40^ Szabó *et al* ^43^ also revealed that the percentage of plasmablasts (CD19^+^CD38^hi^CD27^hi^) is significantly increased in the peripheral blood of patients with SLE, exhibiting a SLEDAI score greater than 6 points. Arce *et al* ^22^ showed that, in patients with SLE, the plasma cell precursor subset (CD19^+^CD27^+/++^CD38^++^) tended to increase compared to that in the controls; however, there were no significant differences in the ratio of B cells (CD19^+^CD27^‐^CD38^∓^) to M‐Bs (CD19^+^CD27^+^CD38^‐^). Our results are in line with their findings. The increased plasmablasts in the peripheral blood of patients with SLE are closely related to the development and clinical manifestations of SLE. Consistently, the frequency of the plasmablast subset decreased in patients with SLE undergoing treatment.

In humans, based on CD19 and CD27 expression, B cells can be distinguished into CD19^+^CD27^‐^ and CD19^+^CD27^+^ B cells. According to IgM and IgD expression, M‐Bs can be divided into two categories: IgM‐positive M‐Bs (CD19^+^CD27^+^IgM^+^IgD^+^)^44^ and CSM‐Bs (CD19^+^CD27^+^IgM^‐^IgD^‐^), accounting for approximately 50%, respectively. Thus, according to the expression of CD19, CD27 and IgD,^40^ several subtypes of B cells can be distinguished from M‐Bs in the antigen‐dependent differentiation stage.^45^, ^46^ NSM‐B is a peculiar type of M‐B that undergoes high‐frequency mutations without class switching recombination in somatic cells.^47^ In contrast, CSM‐B undergoes the above‐mentioned processes in the germinal centre of lymph nodes itself and is, therefore, considered as the traditional M‐B. Szabó *et al* ^43^ demonstrated that the level of DN‐B significantly increased, whereas the frequency of NSM‐B significantly decreased, in SLE. In addition, some studies have shown decreased circulating CD27^+^ M‐Bs and increased circulating CD27^‐^ N‐Bs,^45^, ^48^ while others have shown the opposite in patients with SLE.^22^, ^39^, ^40^, ^49^, ^50^, ^51^ At the antigen‐independent differentiation stage, we noted a slight increasing trend in the N‐B subset in patients with SLE, which was similar to that in the antigen‐dependent stage; however, these differences were not statistically significant. We also reported a decreased frequency of the NSM‐B subset and increased frequency of the DN‐B subset in the peripheral blood of patients with SLE, corroborating the results of other studies.^40^, ^43^ Furthermore, some studies have shown that the deficiency of the NSM‐B subset contributes to viral and bacterial infections.^49^, ^52^ Therefore, we speculate that the decreased frequency of NSM‐B may be related to immune system dysfunction and could explain the high susceptibility of patients with SLE to infection. CSM‐Bs, which are usually referred to as M‐Bs, also significantly decreased in the newly diagnosed patients with SLE, which was comparable to the variation of M‐Bs detected during antigen‐dependent differentiation. However, this difference was not significant in the other groups of patients with SLE. Similarly, we have been suggested that the decrease in the distribution of the CSM‐B subset may be caused by an increased conversion to plasma cells.

DN‐Bs are a category of M‐Bs with unknown function and devoid of CD27 expression, which are phenotypically similar to N‐Bs.^53^ The proportion of CD27^‐^IgD^‐^ B cells is elevated in patients with SLE, further indicating that this increase is associated with disease activity and the presence of specific antibodies such as anti‐dsDNA, anti‐Smith (Sm) and anti‐ribonucleoprotein (RNP and 9G4) antibodies.^40^, ^43^, ^54^ In addition, Anolik *et al* ^50^ showed that the levels of circulating CD27^‐^IgD^‐^ B cells with memory‐like cell characteristics increase in patients with SLE because of a connection between the degree of increase and the high titres of autoantibody, suggesting that DN‐Bs may participate in autoantibody response directly or indirectly. In this study, we found that the proportion of the DN‐B subset increased in SLE and may be related to autoantibody abnormalities, whereas previous studies that focused on this aspect did not show consistent results. Possible reasons for these discrepancies may be the ethnical heterogeneity of the enrolled patients and inconsistency in the selection of B‐cell surface markers.

In conclusion, this study comprehensively analysed the altered frequency of peripheral B‐cell subsets and their correlation with disease activity in patients with SLE. Increased understanding of the differences in B‐cell subsets in patients with SLE is likely to advance our knowledge of the pathogenesis of this disease.

## CONFLICT OF INTEREST

The authors declare no competing interests.

## AUTHORS’ CONTRIBUTIONS

Yanxia Peng: Conceptualization (equal); Data curation (equal); Methodology (equal); Writing‐original draft (equal). Fengbiao Guo: Conceptualization (equal); Data curation (equal); Methodology (equal); Writing‐original draft (equal). Shuzhen Liao: Conceptualization (equal); Data curation (equal); Methodology (equal); Writing‐original draft (equal). Huanjin Liao: Data curation (equal); Methodology (equal). Haiyan Xiao: Writing‐review & editing (equal). Lawei Yang: Writing‐review & editing (equal). Hua‐feng Liu: Supervision (equal). Qingjun Pan: Conceptualization (equal); Data curation (equal); Funding acquisition (equal); Methodology (equal); Writing‐review & editing (equal). QP, YP, FG and SL: The idea generation; experiment conception; and experiment design. SL, YP, SL, HL (Huanjin Liao) and FG: Experiment performance and data analysis. QP, YP, SL, HX and LY: Manuscript writing. HL (Hua‐feng Liu): Study supervision. All authors: The final manuscript revision and manuscript approval.
